# Syntheses, Crystal Structures, and Catalytic Properties of Three Cu(II) and Cobalt(II) Coordination Compounds Based on an Ether-Bridged Tetracarboxylic Acid

**DOI:** 10.3390/molecules28196911

**Published:** 2023-10-02

**Authors:** Xiuqi Kang, Hongyu Wang, Zhenzhong Mei, Xiaoxiang Fan, Jinzhong Gu

**Affiliations:** College of Chemistry and Chemical Engineering, Lanzhou University, Lanzhou 730000, China; 220220923660@lzu.edu.cn (X.K.); hywang@lzu.edu.cn (H.W.); meizhzh19@lzu.edu.cn (Z.M.); fanxx21@lzu.edu.cn (X.F.)

**Keywords:** coordination polymers, crystal structures, new topology, catalysis, cyanosilylation reaction

## Abstract

Three new products, [Cu_2_(*μ*_3_*-*dppa)(2,2′-bipy)_2_(H_2_O)]*_n_*·2*n*H_2_O (**1**), [Co_4_(*μ*_4_*-*dppa)_2_(phen)_4_(H_2_O)_4_]·2H_2_O (**2**), and [Co_2_(*μ*_6_*-*dppa)(*μ-*4,4′-bipy)(H_2_O)_2_]*_n_*·3*n*H_2_O (**3**) were synthesized using a hydrothermal method from Cu(II) and Co(II) metal(II) chlorides, 3-(3,4-dicarboxyphenoxy)phthalic acid (H_4_dppa), and different auxiliary ligands, namely 2,2′-bipyridine (2,2′-bipy),1,10-phenanthroline (phen), and 4,4′-bipyridine (4,4′-bipy). Products **1**–**3** were characterized by elemental analysis, FTIR, TGA, PXRD, SEM, and single-crystal X-ray crystallography. The structure of **1** features a 1D chain of the 2C1 topological type. Compound **2** shows a discrete tetrameric complex. Product **3** demonstrates a 3D metal–organic framework (MOF) with the new topology. Their structure and topology, thermal stability, and catalytic activity were studied. In particular, excellent catalytic activity was demonstrated for copper(II)-polymer **1** in the cyanosilylation reaction at 35 °C.

## 1. Introduction

In recent years, designing and assembling new transition metal coordination polymers and derived materials have become the subjects of extensive investigation owing to their intriguing structures and their various applications as potential functional materials [1,2,3,4,5,6,7,8,9,10,11,12]. The selection of transition metal ions and their compounds is primarily driven by their advantageous characteristics, including cost-effectiveness, availability, as well as their remarkable bio- and photoactive properties [13,14,15]. Moreover, transition metals exhibit a rich and intricate coordination chemistry, allowing for a wide range of ligand types to be employed [1,5,9]. Among the various types of ligands, aromatic polycarboxylic acids have emerged as the most prevalent linkers for the fabrication of coordination polymers and their derived materials [4,10,13]. The prominence of aromatic polycarboxylate ligands lies in their exceptional suitability and effectiveness in the fabrication of coordination polymers, such as thermal stability, various kinds of co-ordination modes, and adjustable level of deprotonation. Currently, research efforts are predominantly focused on the development of novel carboxylate linkers possessing multiple potential coordination sites. Subsequently, their applications in the synthesis of coordination polymers are thoroughly investigated, aiming to create innovative and functional metal–organic architectures.

In organic synthesis, cyanosilylation is classified as one of the fundamental reactions for C−C bond formation [16,17]. This reaction is employed for the synthesis of cyanohydrins, which serve as crucial precursors in the production of high-value fine chemicals and pharmaceuticals [18,19,20,21]. Notably, the catalytic role of coordination polymers in cyanosilylation reactions has attracted considerable attention in scientific investigations [22,23,24]. This prominence stems from the manifold advantages associated with coordination compound catalysts, including their superior catalytic activity, remarkable stability, cost-effectiveness, and remarkable substrate versatility [20,21,22,23,24].

**Scheme 1:**
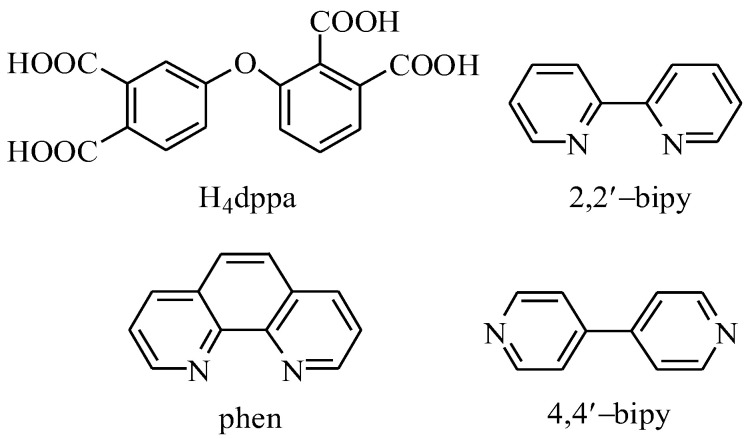
Structural formulae of H_4_dppa and auxiliary ligands.

Following our consistent research path, we have chosen purchasable polycarboxylic acid ligands, which are rarely used in the synthesis of functional coordination polymers [10,13,25,26]. In the current study, we selected an ether-bridged tetracarboxylic acid: 3-(3,4-dicarboxyphenoxy)phthalic acid (H_4_dppa, Scheme 1). This organic acid can serve as a potential semi-flexible ligand for the fabrication of coordination polymers. We chose this carboxylic acid for the synthesis of the coordination polymers for the following reasons: (1) the ligand contains two benzene rings connected by an ether oxygen group, which can undergo subtle structural changes based on the coordination characteristics of metal ions. (2) This ligand contains nine potential coordination sites coming from eight carboxyl oxygen atoms and one ether oxygen atom, leading to multiple coordination modes and promoting the formation of coordination polymers with high-dimensional structures. (3) In 2020, several Mn(II)-dppa and Cd(II)-dppa coordination polymers were reported [27,28]. Although their magnetism and luminescence were researched, their potentialcatalytic activity were neglected. Thus, the present study gave us a good opportunity to develop this field.

Therefore, we designed and synthesized three Cu(II) and Co(II) coordination compounds based on an ether-bridged tetracarboxylate ligand. In this article, we report the preparation, crystal structures, and catalytic properties of three Cu(II) and Co(II) co-ordination compounds constructed from the ether-bridged tetracarboxylate ligand.

## 2. Results and Discussion

### 2.1. Hydrothermal Synthesis of Coordination Polymers

**Scheme 2:**
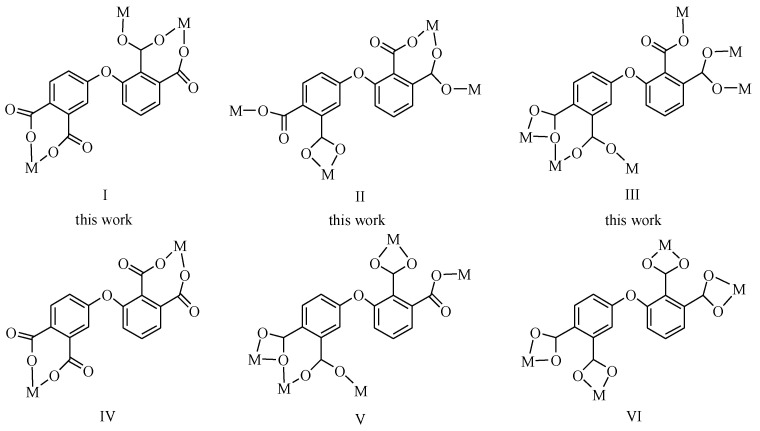
The coordination fashions of dppa^4−^ in this study and previous reported works.

To investigate the role of 3-(3,4-dicarboxyphenoxy) phthalic acid (H_4_dppa) as a ligand in the preparation of coordination polymers, we conducted some hydrothermal reactions using Cu(II) or Co(II)-chlorides, H_4_dppa, NaOH, and auxiliary ligands (2,2′-bipy, phen, or 4,4′-bipy) as reactants. These reactions were performed at 150 °C for 72 h, and then the complexes were formed by slowly cooling the reaction mixture and crystallization. Three experiments have achieved positive results, successfully generating three new coordination compounds and comprehensively characterizing them (see Table 1). The products were collected in the form of microcrystalline solids and characterized in detail through elemental analysis, FTIR, PXRD, TGA, SEM and single-crystal X-ray diffraction techniques. The different structural types shown in **1**–**3** can be ascribed the distinct coordination fashions of metal (II) centers and the presence of different auxiliary ligands. In compounds **1**–**3**, the dppa^4−^ ligand serves as a µ_3_-. µ_4_- or µ_6_-spacer (Scheme 2).

### 2.2. Crystal Structure of ***1***

**Figure 1:**
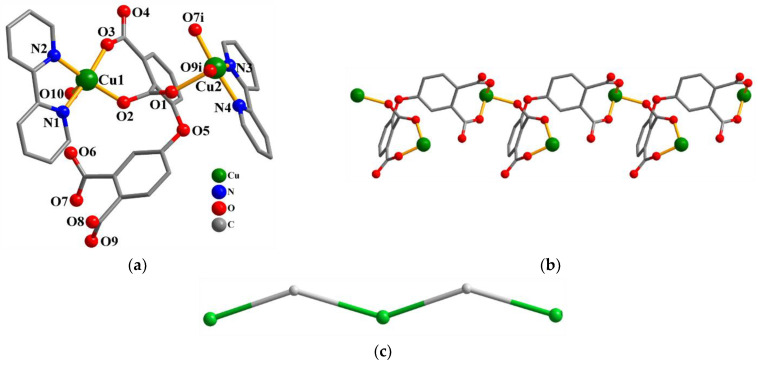
Structure of **1**. (**a**) Coordination environment of Cu(II) atoms; hydrogen atoms are removed. Symmetry code: i = *x*−1, *y*, *z*. (**b**) The 1D chain (view along the *b* axis). (**c**) Topological representation of a 1D chain with a 2C1 topology; view along the *b* axis; 2-connected Cu1 nodes (green), centroids of 2-connected µ_3_-dppa^4−^ nodes (gray).

Compound **1** crystallizes in the triclinic system, space group *P*–1. As shown in Figure 1a, there are two Cu(II) atoms (Cu1 and Cu2), one *μ*_3_*-*dppa^4−^ block, two 2,2′-bipy supporting ligands, one coordinated water molecule, and two free water molecules in the asymmetric unit of compound **1**. The five-coordinate Cu1 center displays a distorted trigonal bipyramidal {CuN_2_O_3_} environment filled by two carboxylate O atoms from one *μ*_3_*-*dppa^4−^ block, one O atom from the coordinated water molecule, and two N donors of the 2,2′-bipy moiety. The Cu2 center is also five-coordinated and features a distorted trigonal bipyramidal {CuN_2_O_3_} geometry that is taken by three carboxylate O atoms from two different *μ*_3_*-*dppa^4−^ blocks and two N atoms from the 2,2′-bipy moiety. The bond distances of Cu–O and Cu–N are 1.927(2)–2.249(2) and 1.992(3)–2.010(3) Å, respectively; these are within the normal ranges observed in related Cu(II) compounds [15,29]. In **1**, the dppa^4−^ ligand adopts the coordination mode I (Scheme 2) with four COO^−^ groups being monodentate or bridging bidentate. The *μ*_3_*-*dppa^4−^ blocks connect adjacent Cu atoms to give a 1D chain (Figure 1b). This 1D chain is described as a uninodal net, defined as a **2C1** topology (Figure 1c).

### 2.3. Crystal Structur of ***2***

**Figure 2:**
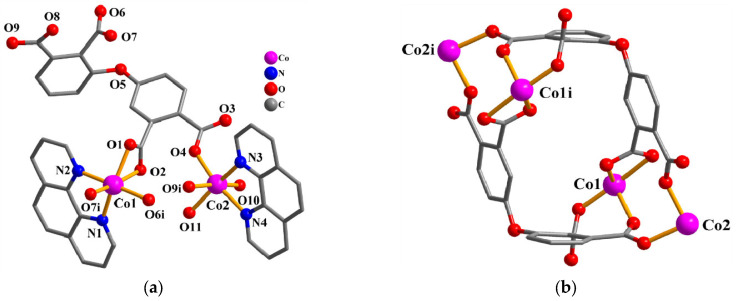
Structure of **2**. (**a**) Coordination environment of Co(II) centers; hydrogen atoms are removed. Symmetry code: i = −*x* +2, −*y* +1, −*z* +1. (**b**) Co_4_ molecular unit; the phen moieties are removed. Symmetry code: i = −*x* +2, −*y* +1, −*z* +1.

The asymmetric unit of **2** consists of two Co(II) centers (Co1 and Co2), a *μ*_4_*-*dppa^4−^ block, two phen-supporting ligands, two coordinated H_2_O, and a free H_2_O. As depicted in Figure 2a, Co1 center is hexa-coordinated and shows a distorted octahedral {CoN_2_O_4_} environment. It is taken by four carboxylate O atoms from two individual *μ*_4_-dppa^4−^ blocks and a pair of N_phen_ atoms. Co2 center is also hexa-coordinated and features a distorted octahedral {CoN_2_O_4_} environment, which is taken by two O atoms from two different*μ*_4_-dppa^4−^ blocks, two O atoms from two coordinated H_2_O, and two N_phen_ donors. The bond distances of Co–O are in the 1.995(3)–2.291(3) Å range, while the Co–N bonds are 2.103(3)–2.148(4) Å, which are comparable to those reported in other Co(II) compounds [10,25]. In **2**, the dppa^4−^ block acts as a *μ*_4_-linker (mode II, Scheme 2), in which four COO^−^ groups show monodentate, bidentate, or bridging bidentatecoordination fashions. The *μ*_4_-dppa^4−^ blocks interconnect the neighboring Co centers to generate a Co_4_ molecular unit (Figure 2b).

### 2.4. Crystal Structure of ***3***

**Figure 3:**
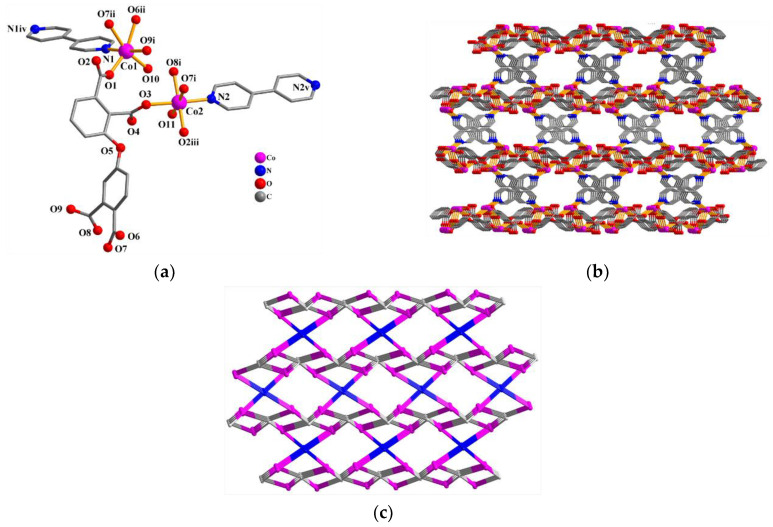
Structure of **3**. (**a**) Coordination environment of Co(II) atoms; hydrogen atoms are removed. Symmetry codes: i =*x*−1, *y*, *z*; ii =*x*−1, −*y* +1/2, *z* +1/2; iii = *x*, −*y* +1/2, *z*−1/2; iv = −*x* +1, −*y*, −*z* +2; v =−*x*, −*y*, −*z*. (**b**) 3D metal–organic framework (view along the *a* axis). (**c**) Topological representation of a 3-nodal net framework displaying a new topology 4-connected Co nodes (pink), centroids of 6-connected *µ*_6_-dppa^4−^ nodes (gray), and centroids of 2-connected *µ*-4,4′-bipy linkers (blue).

The asymmetric unit of Co(II) compound **3** bears two Co(II) atoms (Co1 and Co2), one *µ*_6_-dppa^4−^ block, one *µ*-4,4′-bipy auxiliary ligand, two coordinated H_2_O, and three free H_2_O (Figure 3a). Both Co(II) centers adopt distorted octahedral {CoNO_5_} environments taken by four carboxylate O atoms from three independent *µ*_6_-dppa^4−^ blocks, one O atom coming from the coordinated water molecule, and a N_4,4′-bipy_ donor. The Co–O [1.987(4)–2.246(4) Å] and Co–N [2.114(5)–2.148(5) Å] bonds are within standard values [15,29,30]. The dppa^4−^ block acts as a *µ*_6_-linker (mode III, Scheme 2) with four COO^−^ groups being monodentate, bridging bidentate, or tridentate. The 4,4’*-*bipy auxiliary ligand shows a bridging coordination fashion. The *µ*_6_-dppa^4−^ blocks and *µ*-4,4′-bipy moieties linked Co(II) centers to form a 3D metal–organic framework (Figure 3b). The 3D framework in this structure is built from the four-linked Co, six-linked *µ*_6_-dppa^4−^ nodes, and two-linked *µ*-4,4′-bipy nodes (Figure 3c). It can be defined as a three-nodal net with a new topology and point symbol of (4^3^. 8^2^.10)_2_(4^6^.6^6^.8^3^)(8).

### 2.5. TGA and PXRD Data

The thermogravimetric analysis (TGA) was performed on crystalline samples of compounds **1**–**3** to investigate the thermal stability of three compounds in the range of room temperature to 800 °C under nitrogen gas flow (Figure S2). For compound **1**, the weight loss in the range of 49–138 °C corresponds to the departure of two lattice water molecules and one H_2_O ligand (observed 6.4%, calculated 6.5%), followed by decomposition above 196 °C. For **2**, the weight loss corresponding to the release of two lattice water molecules and four H_2_O ligands is observed from 53 to 210 °C (observed, 6.1%; calculated, 6.2%). The decomposition of the anhydrous compound occurs at 260 °C. The TGA plot of compound **3** shows that the weight loss from 42 to 175 °C attributes to the loss of three free and two coordinated water molecules (observed, 12.4%; calculated, 12.7%). Then, the compound reaches a plateau with no further weight loss up to 246 °C. Upon further heating, the host framework begins to decompose. The corresponding metal oxides (Cu_2_O and CoO) are expected as a final product of compounds **1**–**3**. A summary of the TGA data is provided in Table S1.

In order to confirm the phase purity of compounds **1**–**3**, the PXRD (powder X-ray diffraction) experiments were performed on the as-prepared crystalline solids of compounds **1**–**3**. As illustrated in Figure S3, the main peaks of experimental spectra of compounds **1**–**3** very well match the diffraction peaks calculated on the basis of single-crystal X-ray diffraction data. The results confirm the phase purity of compounds **1**–**3** obtained by the hydrothermal synthesis.

### 2.6. Crystal Surface Morphology Analysis

The field emission scanning electron microscope (SEM) is used to observe the surface morphology of coordination compounds **1**–**3**. As shown in Figure S4, compound **1** has a long needle structure with non-directional distribution, the outer surface is smooth without roughness, and the length is different. Compound **2** has a block structure with non-directional distribution, the outer surface is smooth, and the sizes are different. Compound **3** has a long block structure, the surface is smooth, the sizes are the same, and they are stacked together. 

### 2.7. Catalytic Cyanosilylation of Aldehydes with TMSCN

**Scheme 3:**
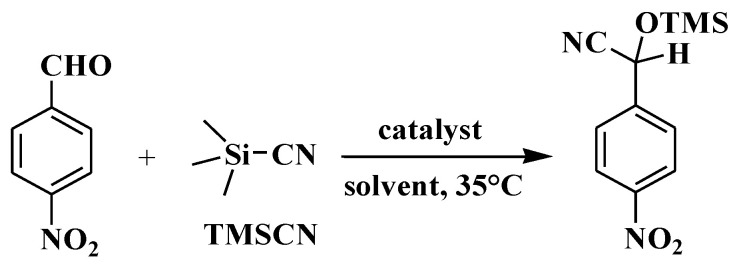
Cyanosilylation of model substrate (4-nitrobenzaldehyde).

Based on the catalytic function of some coordination polymers in the silicocyanide reaction between aldehydes and TMSCN [20,21,22,23], we tested the catalytic activities of compounds **1**–**3** as heterogeneous catalysts for the silicocyanide reaction between 4-nitrobenzaldehyde and TMSCN, with the aim of screening potential excellent catalysts. The catalytic reactions were performed in dichloromethane as a solvent at 35 °C, and the final results showed that 2-(4-nitrophenyl)-2-[(trimethylsilyl)oxy]acetonitrile (Scheme 3, Table 1) was obtained. We conducted detailed experiments on the effects of reaction time, solvent type, and catalyst dosage on the yield, as well as the applicability of substrates.

The results showed that compound **1** gave the highest activity (100% conversion, Table 1). We chose it as the preferable catalyst for further optimization research. Specifically, the reaction time has a significant impact on the yield. We found that, when the reaction time increases from 1 h to 10 h, the yield of the product increases from 38% to 100% (Table 1, entries 1–6; Figure S5). At the same time, we also investigated the effect of catalyst dosage. When the catalyst dosage increased from 2 mol% to 3 mol%, the yield of the product increased from 94% to 100% (entries 6 and 7). In addition, the effect of solvents such as acetonitrile, methanol, tetrahydrofuran, and chloroform was also tested. The experimental results demonstrated they gave lower yields of the products (84% to 98%).

The experimental results show that the conversion efficiency of the silicocyanidation reaction of 4-nitrobenzaldehyde is very low (only 5% yield) without a catalyst (entry 14, Table 1). When carboxylic acid ligands or copper chloride are used, the yields are 6% and 8%, respectively (entries 15 and 16, Table 1). Although the relationship between the structure and activity of the catalyst is not very clear in this work, the higher activity of complex **1** may be related to its unsaturated coordination sites and more accessible active sites [31,32].

The substrate range of benzaldehyde with different substituents was studied using the optimized cyanosilylation reaction parameters (3.0 mol% **1**, CH_2_Cl_2_, 10 h), and the corresponding product yields ranged from 28% to 100% (Table S2). Benzaldehyde with electron withdrawing groups (e.g., –NO_2_ or –Cl) as a substrate exhibits high yield, which is due to the increased electrophilicity of the substrate by these groups. Benzaldehyde containing electron-donating groups (e.g., –CH_3_ or –OCH_3_) as a substrate exhibits lower yields (entries 7 and 8, Table S2).

Finally, cyclic catalytic experiments were conducted on catalyst **1**, and the results showed that compound **1** still maintained its activity after at least five cycles of catalytic cycling (Figure S6). Moreover, the powder diffraction pattern determined that the structure of compound **1** remained unchanged after the catalytic reaction (Figure S7), although several new peaks or widening of the original peaks were observed. These changes may be related to the decrease in crystallinity of the catalyst after cyclic catalysis and the presence of impurities.

The catalytic activity of compound **1** in the cyanosilylation reaction of 4-nitrobenzaldehyde with TMSCN is equivalent to [33,34] or slightly higher [35,36,37,38,39] than that of other coordination containing carboxylate ligands (Table S3).

## 3. Experimental

### 3.1. Materials and Measurements

Except where otherwise noted, all chemicals were commercially available and were used as received. H_4_dppa was acquired from Yanshen Tec. Co., Ltd. (Jilin, China). Bruker EQUINOX 55 spectrometer (Bruker Corporation, Billerica, MA, USA) was used to record the FTIR spectra (KBr discs). ElementarVario EL elemental analyzer (Elementar, Langenselbold, Germany) was utilized to obtain the C/H/N elemental analyses (EA). LINSEIS STA PT1600 thermal analyzer (LinseisMessgeräte GmbH, Selb, Germany) was used to record thermogravimetric analyses (TGA) under N_2_ flow with a heating rate of 10 °C/min.Rigaku-Dmax 2400 diffractometer (Cu-Kα radiation; *λ* = 1.54060 Å, Rigaku Corporation, Tokyo, Japan) was used to run the powder X-ray diffraction patterns (PXRD) of the obtained compounds. CDCl_3_ was used as the solvent for ^1^H NMR measurements on a JNM ECS 400M spectrometer (Bruker BioSpin AG, Fällanden, Switzerland). Scanning electron microscopy (SEM) images were taken on a field emission scanning electron microscope (S-4800, HITACHI, Tokyo, Japan).

### 3.2. Synthesis of [Cu_2_(μ_3_-dppa)(2,2′-bipy)_2_(H_2_O)]_n_·2nH_2_O (***1***)

CuCl_2_·2H_2_O (34.0 mg, 0.2 mmol), H_4_dppa (34.6 mg, 0.1 mmol),2,2′-bipy (31.2 mg, 0.2 mmol), and NaOH (16.0 mg, 0.4 mmol) were mixed in H_2_O (10 mL) and stirred for 15 min in a vial at room temperature. Then, it was sealed in a 20 mL Teflon-lined bomb and heated at 150 °C for 72 h. After the bomb was cooled down to room temperature, the bomb was opened. Some green needle-shaped crystals of compound **1** were picked out manually, washed with water, and dried (yield: 49% based on H_4_dppa). Calculated for C_36_H_28_Cu_2_N_4_O_12_: C 51.74, N 6.70, H 3.38%. Found: C 51.96, N 6.68, H 3.40%. FTIR (KBr, cm^−1^): 3453 m, 3113 w, 1611 s, 1580 s, 1552 s, 1473 w, 1447 w, 1388 s, 1313 w, 1272 w, 1237 w, 1161 w, 1062 w, 1032 w, 984 w, 943 w, 894 w, 864 w, 836 w, 774 w, 730 w, 663 w.

### 3.3. Synthesis of [Co_4_(μ_4_-dppa)_2_(phen)_4_(H_2_O)_4_]·2H_2_O (***2***)

CoCl_2_∙6H_2_O (47.4 mg, 0.2 mmol), H_4_dppa (34.6 mg, 0.1 mmol), phen (39.6 mg, 0.2 mmol), and NaOH (16.0 mg, 0.4 mmol) were mixed in H_2_O (10 mL) and stirred for 15 min in a vial at room temperature. Then, it was sealed in a 20 mL Teflon-lined bomb and heated at 150 °C for 72 h. After the bomb was cooled down to room temperature, the bomb was opened. Some orange block-shaped crystals of compound **2** were picked out manually, washed with water, and dried (yield: 52% based on H_4_dppa). Calculated for C_40_H_28_Co_2_N_4_O_12_: C 54.94, N 6.41, H 3.23%. Found: C 55.18, N 6.42, H 3.21%. FTIR (KBr, cm^−1^): 3616 w, 3365 m, 3060 w, 1553 s, 1516 w, 1424 w, 1398 s, 1300 w, 1254 w, 1232 w, 1215 w, 1139 w, 1103 w, 1059 w, 979 w, 850 w, 814 w, 782 w, 725 w, 694 w, 644 w.

### 3.4. Synthesis of [Co_2_(μ_6_-dppa)(μ-4,4΄-bipy)(H_2_O)_2_]_n_·3nH_2_O (***3***)

Compound **3** was synthesized following a procedure similar to **2,** except using 4,4′-bipy (31.2 mg, 0.2 mmol) instead of phen. Some pink block-shaped crystals of compound **3** were collected (yield: 53% based on H_4_dppa). Calculated for C_26_H_24_Co_2_N_2_O_14_: C 44.21, N 3.97, H 3.42%. Found: C 44.47, N 3.94, H 3.44%. FTIR (KBr, cm^−1^): 3404 m, 1607 s, 1557 s, 1406 s, 1299 w, 1153 w, 1072 w, 996 w, 903 w, 866 w, 809 w, 770 w, 704 w, 672 w, 632 w.

### 3.5. Single-Crystal X-ray Diffraction and Topological Analysis

We collected the crystal data of compounds **1**–**3** on a Bruker APEX-II CCD diffractometer (graphite-monochromated Mo/Cu*K*_α_ radiation; *λ* = 0.71073/1.54178/Å). The structures were solved by direct methods and refined by full-matrix least-squares on *F*^2^ with SHELXS-97 and SHELXL-97 [40]. The carbon, oxygen, and nitrogen atoms were refined anisotropically using full-matrix least-squares methods on *F^2^*. By using riding models, the hydrogen atoms were placed in calculated positions. In **3**, the pyridine ring (N1, C22–C26) of 4,4′-bipy ligand was split over two sites and refined with 0.60 and 0.40 occupancies. The details of crystal data and refinement parameters of compounds **1**–**3** are presented in Table 2. The important bond lengths and angles are listed in Tables S4 and S5 (Supplementary Materials). CCDC 2274892–2274894 contain the supplementary crystallographic data of **1**–**3**.

For the topological analysis of metal–organic networks, the concept of the underlying (simplified) networks was adopted [41,42]. These simplified networks were obtained by removing terminal ligands and converting all the bridging ligands into the respective centroids (their connectivity was retained).

### 3.6. Catalytic Activity in Cyanosilylation of Benzaldehydes

A mixture of 4-nitrobenzaldehyde (0.50 mmol), trimethylsilyl cyanide (TMSCN, 1.0 mmol), and catalyst (typically 3.0 mol%) was placed into a glass tube, and then CH_2_Cl_2_ (2.5 mL) was added. The mixture was capped, stirred, and heated at 35 °C for a desired time. Then, the solid catalyst was removed through centrifugation. The remaining solution was evaporated under vacuum to give a solid crude product. The residue was dissolved in CDCl_3_. Quantitative analysis of the product was performed using ^1^H NMR spectroscopy (JNM ECS 400M spectrometer, Jeol Ltd., Tokyo, Japan) (see Figure S8 for more details). In order to conduct cyclic catalytic experiments, solid catalysts were collected by centrifugation and washed with dichloromethane, dried at room temperature, and then reused in subsequent tests. According to the above procedure, the range of other aldehyde substrates was also studied.

## 4. Conclusions

In summary, we have synthesized three Cu(II) and Co(II) coordination compounds based on an ether-bridged tetracarboxylate ligand. The structural types of compounds **1**−**3** range from 0D molecular tetramers (**2**) to 1D network (**1**) and 3D metal–organic framework (**3**). The catalytic activity of three product**s** were studied. Compound **1** revealed an excellent catalytic activity in the cyanosilylation of aldehydes with TMSCN.

## Figures and Tables

**Table 1:** Cu-catalyzed cyanosilylation of 4-nitrobenzaldehyde with TMSCN.

Entry	Catalyst	Time, h	Catalyst Loading, mol%	Solvent	Yield *^a^*, %
1	**1**	1	3.0	CH_2_Cl_2_	38
2	**1**	2	3.0	CH_2_Cl_2_	67
3	**1**	4	3.0	CH_2_Cl_2_	81
4	**1**	6	3.0	CH_2_Cl_2_	90
5	**1**	8	3.0	CH_2_Cl_2_	97
6	**1**	10	3.0	CH_2_Cl_2_	100
7	**1**	10	2.0	CH_2_Cl_2_	94
8	**1**	10	3.0	CH_3_CN	87
9	**1**	10	3.0	THF	84
10	**1**	10	3.0	CH_3_OH	98
11	**1**	10	3.0	CHCl_3_	96
12	**2**	10	3.0	CH_2_Cl_2_	85
13	**3**	10	3.0	CH_2_Cl_2_	78
14	Blank	10	-	CH_2_Cl_2_	5
15	CuCl_2_·2H_2_O	10	3.0	CH_2_Cl_2_	8
16	H_4_dppa	10	3.0	CH_2_Cl_2_	6

*^a^* Calculated by ^1^H NMR spectroscopy: mol(product)/mol(aldehyde + product) × 100.

**Table 2:** Summary of crystal data for compounds **1**−**3**.

Compound	1	2	3
Chemical formula	C_36_H_28_Cu_2_N_4_O_12_	C_40_H_28_Co_2_N_4_O_12_	C_26_H_24_Co_2_N_2_O_14_
Molecular weight	835.70	874.52	706.33
Crystal system	Triclinic	Triclinic	Monoclinic
Space group	*P*-1	*P*-1	*P*2_1_/*c*
*a*/Å	10.2566(6)	12.6141(11)	11.1877(7)
*b/*Å	11.6991(8)	13.0964(8)	19.1989(13)
*c*/Å	15.1791(13)	13.1405(10)	13.5420(7)
*α/*(°)	84.044(7)	64.646(7)	90
*β*/(°)	80.245(6)	64.704(8)	104.927(6)
*γ/*(°)	74.586(6)	70.835(7)	90
*V*/Å^3^	1727.2(2)	1745.3(3)	2810.6(3)
*Z*	2	2	4
*F*(000)	852	892	1440
Crystal size/mm	0.24 × 0.23 × 0.22	0.23 × 0.22 × 0.20	0.22 × 0.21 × 0.18
*θ* range for data collection	3.295–25.050	3.795–69.942	4.089–69.951
Limiting indices	−12 ≤ *h* ≤ 12, −13 ≤ *k* ≤ 13, −17 ≤ *l* ≤ 18	−15≤ *h* ≤ 15, −11 ≤ *k* ≤ 15, −13 ≤ *l* ≤ 15	−9 ≤ *h* ≤ 13, −20 ≤ *k* ≤ 20, −16 ≤ *l* ≤ 16
Reflections collected/unique (*R*_int_)	6083/4871 (0.0294)	6435/4618 (0.0505)	5238/3248 (0.0738)
*D*_c_*/*(Mg·cm^−3^)	1.607	1.664	1.669
*μ*/mm^−1^	1.304	8.108	9.928
Data/restraints/parameters	6083/0/487	6435/0/523	5238/48/437
Goodness-of-fit on *F*^2^	1.029	1.025	1.031
Final *R* [(*I* ≥ 2σ(*I*))] *R*_1_, w*R*_2_	0.0402, 0.0865	0.0541, 0.1250	0.0634, 0.1301
R (all data) *R*_1_, w*R*_2_	0.0534, 0.0939	0.0833, 0.1446	0.1146, 0.1553
Largest diff. peak & hole/(e·Å^−3^)	0.339 &−0.386	0.811 &−0.458	0.427&−0.505

## Data Availability

Not applicable.

## Supplementary Materials

The following data are available online at https://www.mdpi.com/article/10.3390/molecules28196911/s1. Figure S1: FT-IR spectra, Figure S2: TGA curves, Figure S3: PXRD patterns, Figure S4: SEM images, additional catalysis data (Figures S5–S8), Table S1: TGA data, Table S2: Substrate scope of catalytic reaction, Table S3: Comparison of catalytic activity, Tables S4 and S5: selected bonding and H-bonding parameters for **1**–**3**.

## Sample Availability

Samples of the compounds are available from the authors.
