# Supplementary material for: Syntheses, Crystal Structures, and Catalytic Properties of Three Cu(II) and Cobalt(II) Coordination Compounds Based on an Ether-Bridged Tetracarboxylic Acid
Source: Molecules. 2023 Oct 2;28(19):6911. doi: 10.3390/molecules28196911 (PMC10574591; doi:10.3390/molecules28196911)
Supplement: Supplementary file 1 [file molecules-28-06911-s001.zip › molecules-2612731-supplementary.pdf]

Supplementary Material

Syntheses, Crystal Structures, and Catalytic Properties of Three Cu(II) and Cobalt(II) Coordination Compounds Based on an Ether-bridged Tetracarboxylic Acid

Xiuqi Kang, Hongyu Wang, Zhenzhong Mei, Xiaoxiang Fan, Jinzhong Gu*

College of Chemistry and Chemical Engineering, Lanzhou University, Lanzhou 730000, China; 220220923660@lzu.edu.cn; hywang@lzu.edu.cn; meizhzh19@lzu.edu.cn; fanxx21@lzu.edu.cn

* Correspondence: gujzh@lzu.edu.cn (J.Z.G.); Tel.: +86-931-8915196

Supplementary material contains:

Figure S1. FT-IR spectra of compounds **1**–**3**.

Figure S2. TGA curves for **1**–**3**.

Figure S3. Powder X-ray diffraction for **1**–**3**.

Figure S4. The surface morphology of compounds **1**–**3.**

Figure S5. Accumulation of 2-(4-nitrophenyl)-2-[(trimethylsilyl)oxy]acetonitrile vs. time in the cyanosilylation of 4-nitrobenzaldehyde with TMSCN catalysed by **1**. Reaction conditions are those of Table 2, entries 1−6..

Figure S6. Catalyst recycling experiments in the cyanosilylation of 4-nitrobenzaldehyde with TMSCN catalyzed by **1** (conditions of Table 2, Entry 6).

Figure S7. PXRD patterns for **1**: simulated (red), before (black) and after (blue) catalysis.

Figure S8. Example of integration in the ^{1}H-NMR spectrum for the determination of the cyanosilylation of 4-nitrobenzaldehyde with TMSCN products (Table 1, Entry 6).

Table S1. Summary of thermal analysis data for compounds **1**–**3**.

Table S2 Substrate scope for Cu-catalyzed cyanosilylation of substituted benzaldehydes with TMSCN.

Table S3 Comparison of various catalysts for the cyanosilylation between 4-nitrobenzaldehyde with TMSCN.

Table S4. Selected bond lengths (Å) and bond angles (°) for **1**–**3**.

Table S5 Hydrogen bonds in crystal packing [Å, °] for **1**–**3**.

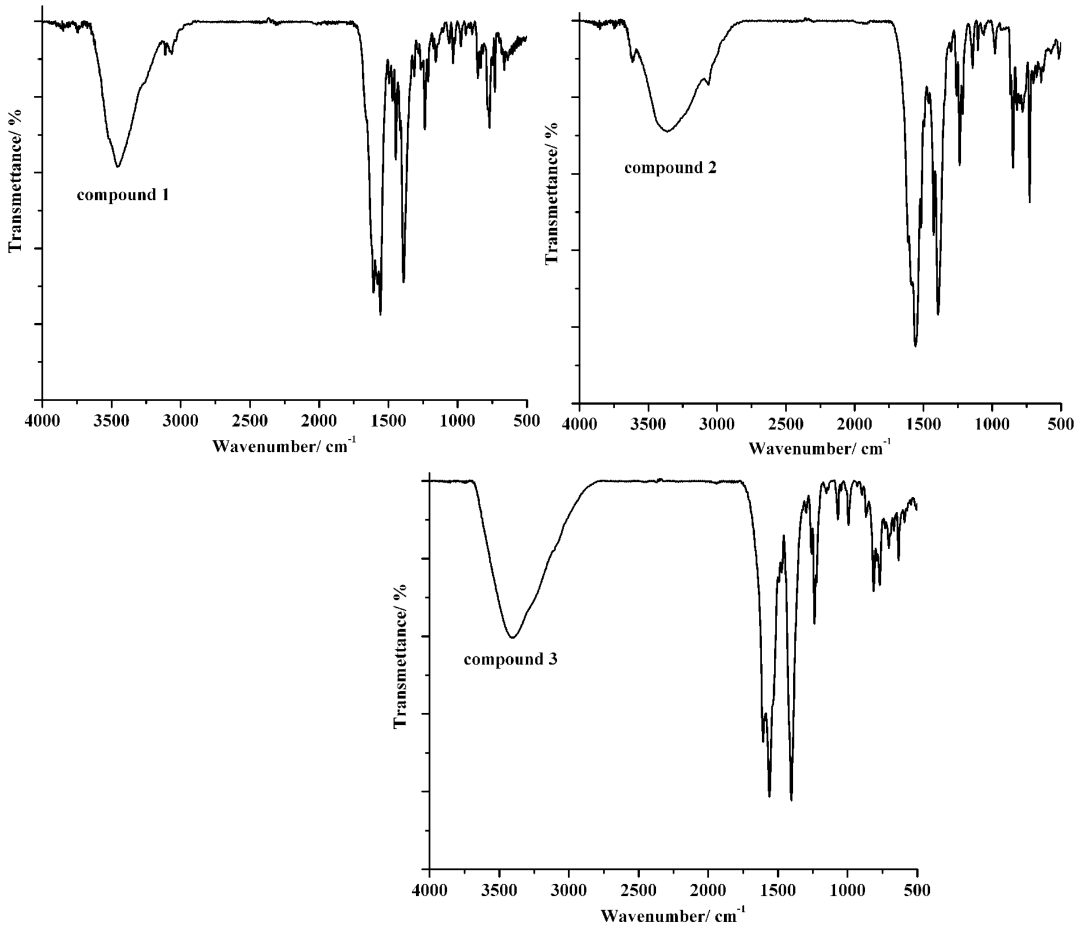

Figure S1. FT-IR spectra of compounds **1–3**.

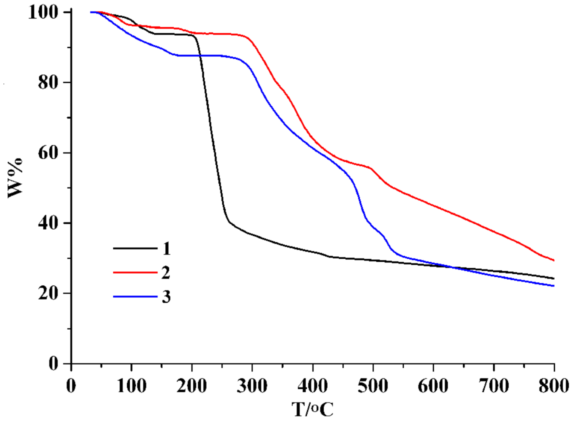

Figure S2. TGA plots of compounds **1–3.**

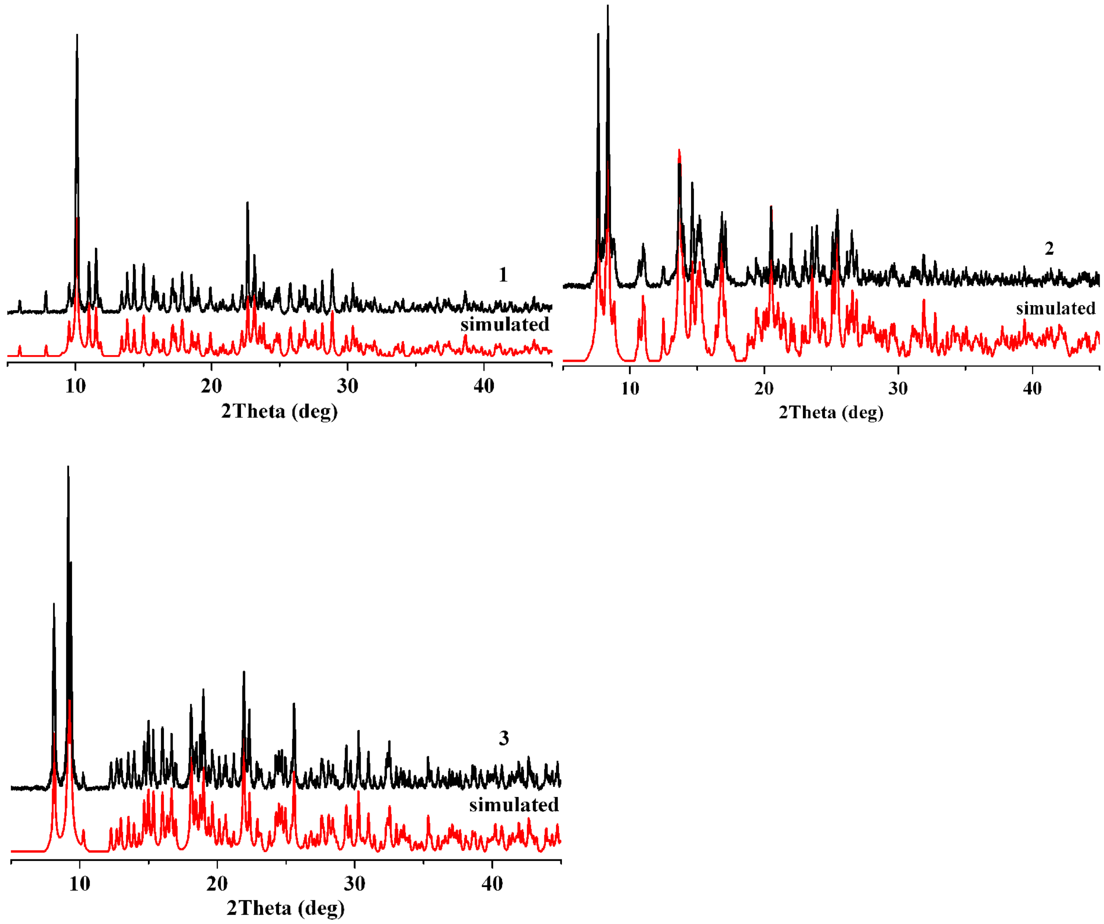

Figure S3. PXRD patterns of compounds **1–3** at room temperature.

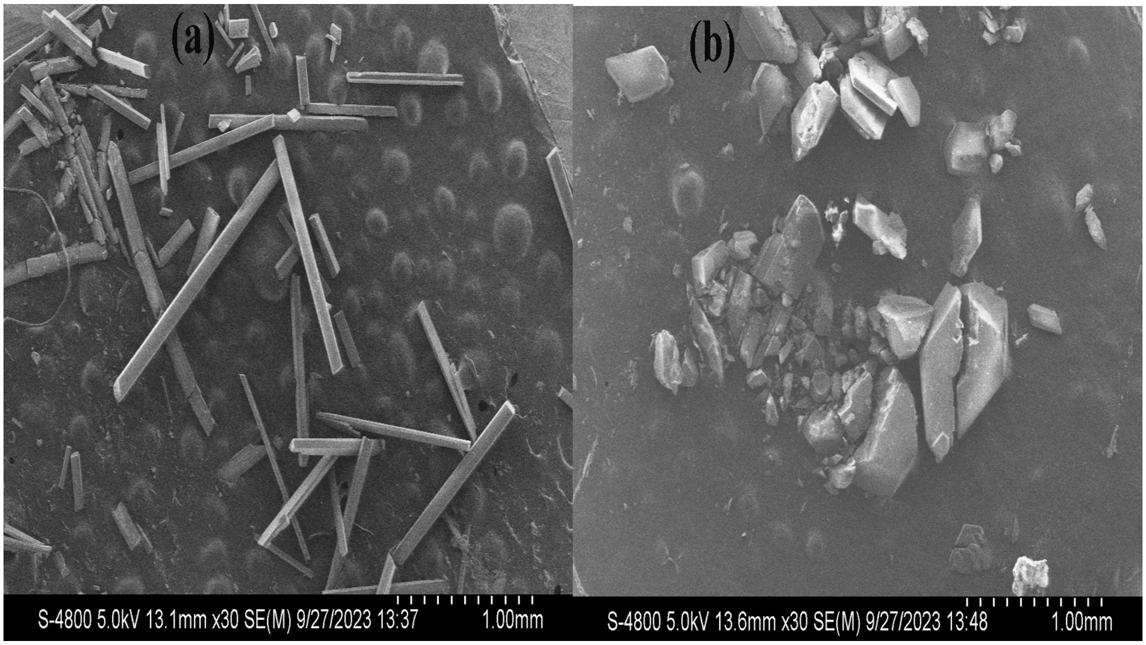

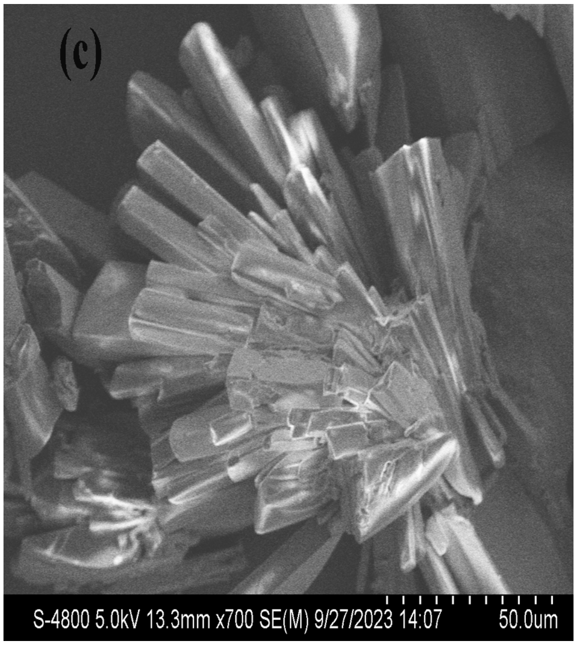

Figure S4. The surface morphology of compound **1** (a), compound **2** (b) and compound **3** (c).

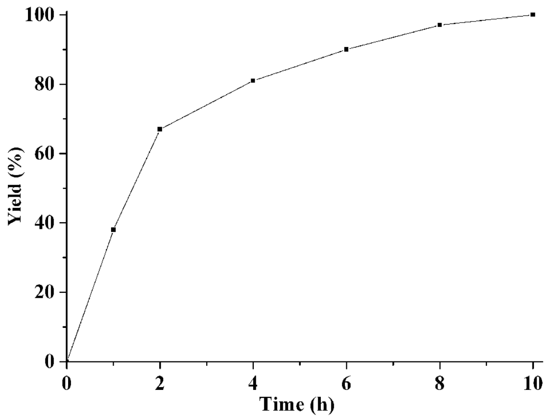

Figure S5. Accumulation of 2-(4-nitrophenyl)-2-[(trimethylsilyl)oxy]acetonitrile vs. time in the cyanosilylation of 4-nitrobenzaldehyde with TMSCN catalysed by **1**. Reaction conditions are those of Table 2, entries 1–6.

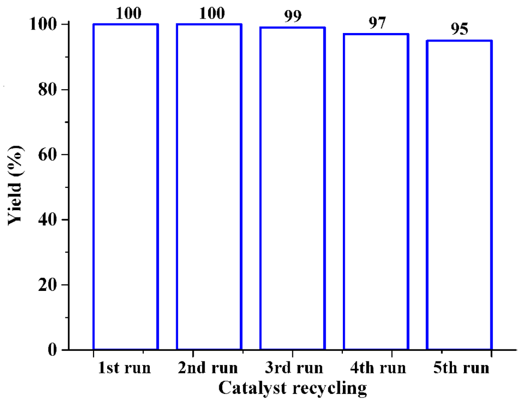

Figure S6. Catalyst recycling experiments in the cyanosilylation of 4-nitrobenzaldehyde with TMSCN catalyzed by **1** (conditions of Table 2, Entry 6).

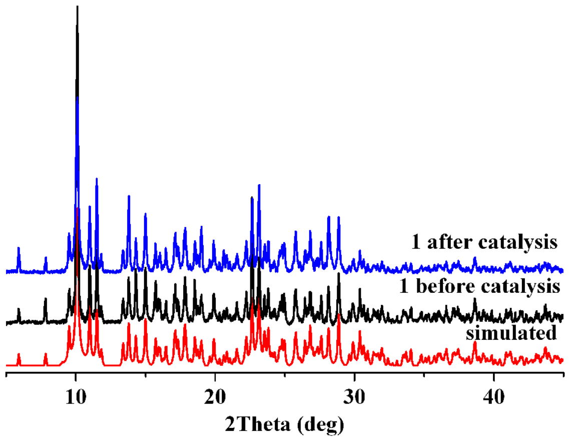

Figure S7. PXRD patterns for **1**: simulated (red), before (black) and after (blue) catalysis.

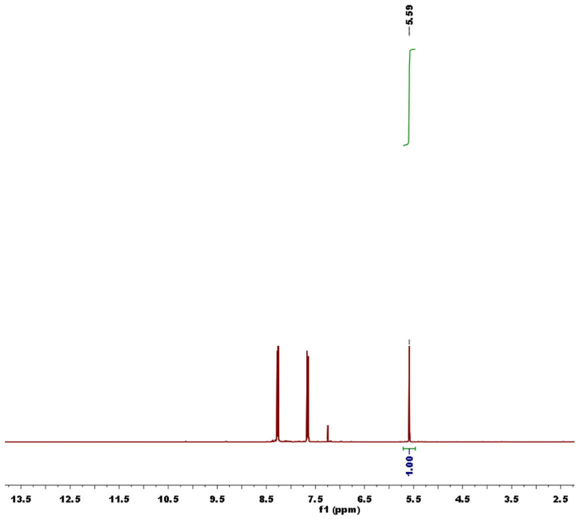

Figure S8. Example of integration in the ^{1}H-NMR spectrum for the determination of the cyanosilylation of 4-nitrobenzaldehyde with TMSCN products (Table 1, Entry 6).

Table S1. Summary of thermal analysis data for compounds **1–3**.

Compound	Dehydration temperature interval (°C)	Dehydration weight loss (%)		Eliminated moieties	Decomposition temperature (°C)	Remaining weight at 800 °C (%)		Remaining residue
		exptl	calcd			exptl	calcd	
**1**	49-138	6.4	6.5	$3H_2O$	>196	24.2	17.1	Cu_2O
**2**	53-210	6.1	6.2	$6H_2O$	>260	29.4	17.1	CoO
**3**	42-175	12.4	12.7	5 H_2O	>246	22.1	21.2	CoO

Table S2. Substrate scope for Cu-catalyzed cyanosilylation of substituted benzaldehydes with TMSCN[a].

Entry	Substituted benzaldehyde substrate ($R-C_6H_4CHO$) su	Product Yield[b], %
1	R = H	97
2	R = 2-NO_2	98
3	R = 3-NO_2	100
4	R = 4-NO_2	100

5	R = 4-Cl	95
6	R = 4-OH	60
7	R = 4-CH_3	57
8	R = 4-OCH_3	28

[a] Reaction conditions: aldehyde (0.5 mmol), TMSCN (1.0 mmol), catalyst **1** (3.0 mol-%), and CH_2Cl_2 (1.0 mL) at 35 ℃. [b] Calculated by ^{1}H NMR spectroscopy.

Table S3. Comparison of various catalysts for the cyanosilylation between 4-nitrobenzaldehyde with TMSCN.

Entry	Catalyst	Catalyst (mol%)	Solvent	Time (h)	Temp. (°C)	Conversion (%)	Ref.
1	$[Cu_2(\mu_3\text{-dppa})(2,2'\text{-bipy})_2(H_2O)]_n\cdot 2nH_2O$	3	CH_2Cl_2	10	35	100	This work
2	$\{Mn_2(bdc)_2(DMF)_2\}_n$	2.5	CH_2Cl_2	19	30	100	29
3	$[Cd_3(tipp)(bpdc)_2]\cdot DMA\cdot 9H_2O$	0.6	Solvent free	18	RT	100	30
4	Zn-ADBA	0.3	CH_2Cl_2	12	25	92.3	31
5	$[Cu_3(OH)_2(L)_2]_n\cdot n(H_2O)$	2	CH_2Cl_2	10	25-30	65	32
6	$[Zn(3,3'\text{-TPDC})]\cdot DMF\cdot 2H_2O$	0.8	toluene	24	50	77	33
7	BINAPDA-Zr-MOF	5	CH_3CN	5	0	85	34
8	Tb-TCA	2	CH_2Cl_2	4	RT	47	35
9	$[Sc(pydc)(H_2O)(NO_3)]$	10	CH_3CN	24	RT	85.2	36

Table S4. Selected bond lengths (Å) and bond angles (°) for **1–3**.

			**1**		
Cu(1)–O(2)	1.931(2)	Cu(1)–O(3)	1.927(2)	Cu(1)–O(10)	2.246(2)
Cu(1)–N(1)	1.992(3)	Cu(1)–N(2)	2.010(3)	Cu(2)–O(1)	2.249(2)
Cu(2)–O(7)i	1.944(2)	Cu(2)–O(9)i	1.927(2)	Cu(2)–N(3)	1.996(3)
Cu(2)–N(4)	2.004(3)				
O(3)–Cu(1)–O(2)	94.64(10)	N(1)–Cu(1)–O(3)	170.98(10)	O(2)–Cu(1)–N(1)	90.55(10)
N(2)–Cu(1)–O(3)	91.18(11)	O(2)–Cu(1)–N(2)	148.34(10)	N(2)–Cu(1)–N(1)	80.58(11)
O(3)–Cu(1)–O(10)	95.07(10)	O(2)–Cu(1)–O(10)	101.38(9)	O(10)–Cu(1)–N(1)	91.12(10)
O(10)–Cu(1)–N(2)	109.07(10)	O(9)i–Cu(2)–O(7)A	91.22(10)	O(9)i–Cu(2)–N(3)	168.56(11)
O(7)i–Cu(2)–N(3)	93.98(10)	O(9)i–Cu(2)–N(4)	93.22(11)	O(7)i–Cu(2)–N(4)	173.88(11)
N(3)–Cu(2)–N(4)	80.91(11)	O(9)i–Cu(2)–O(1)	92.81(9)	O(7)i–Cu(2)–O(1)	92.27(9)
N(3)–Cu(2)–O(1)	97.15(9)	N(4)–Cu(2)–O(1)	91.72(10)		
			**2**		
Co(1)–O(1)	2.125(3)	Co(1)–O(2)	2.291(3)	Co(1)–O(7)i	1.995(3)
Co(1)–O(8)i	2.106(3)	Co(1)–N(1)	2.103(3)	Co(1)–N(2)	2.105(3)
Co(2)–O(4)	2.060(3)	Co(2)–O(9)i	2.144(3)	Co(2)–O(10)	2.135(3)
Co(2)–O(11)	2.132(3)	Co(2)–N(3)	2.129(3)	Co(2)–N(4)	2.148(4)
O(7)i–Co(1)–N(1)	95.69(13)	O(7)i–Co(1)–N(2)	90.73(13)	N(1)–Co(1)–N(2)	78.99(14)
O(7)i–Co(1)–O(8)i	90.47(12)	O(8)i–Co(1)–N(1)	99.69(13)	O(8)i–Co(1)–N(2)	178.30(13)
O(7)i–Co(1)–O(1)	113.70(12)	O(1)–Co(1)–N(1)	149.10(13)	O(1)–Co(1)–N(2)	90.84(13)
O(8)i–Co(1)–O(1)	89.79(12)	O(7)i–Co(1)–O(2)	173.36(12)	O(2)–Co(1)–N(1)	90.91(12)
O(2)–Co(1)–N(2)	89.82(12)	O(8)i–Co(1)–O(2)	89.13(11)	O(1)–Co(1)–O(2)	59.67(11)
O(4)–Co(2)–N(3)	96.82(13)	O(4)–Co(2)–O(11)	91.45(12)	O(11)–Co(2)–N(3)	162.92(14)
O(4)–Co(2)–O(10)	89.80(12)	O(10)–Co(2)–N(3)	108.13(12)	O(10)–Co(2)–O(11)	86.73(12)
O(4)–Co(2)–O(9)i	88.73(12)	N(3)–Co(2)–O(9)i	81.45(12)	O(11)–Co(2)–O(9)i	83.84(12)
O(10)–Co(2)–O(9)i	170.41(11)	O(4)–Co(2)–N(4)	169.91(12)	N(3)–Co(2)–N(4)	77.09(14)
O(11)–Co(2)–N(4)	96.56(13)	O(10)–Co(2)–N(4)	84.56(13)	N(4)–Co(2)–O(9i	98.19(12)
			**3**		
Co(1)–O(1)	1.987(4)	Co(1)–O(6)ii	2.131(4)	Co(1)–O(7)ii	2.246(4)
Co(1)–O(9)i	2.085(4)	Co(1)–O(10)	2.072(4)	Co(1)–N(1)	2.114(5)
Co(2)–O(2)iii	2.070(4)	Co(2)–O(3)	2.131(4)	Co(2)–O(7)i	2.128(4)
Co(2)–O(8)i	2.084(4)	Co(2)–O(11)	2.064(4)	Co(2)–N(2)	2.148(5)
O(1)–Co(1)–O(10)	98.11(17)	O(1)–Co(1)–O(9)i	93.19(17)	O(9)i–Co(1)–O(10)	90.85(17)
N(1)–Co(1)–O(1)	91.20(18)	N(1)–Co(1)–O(10)	91.37(19)	O(9)i–Co(1)–N(1)	174.76(18)
O(1)–Co(1)–O(6)ii	158.81(17)	O(10)–Co(1)–O(6)ii	103.07(17)	O(6)ii–Co(1)–O(9)i	87.14(17)
O(6)ii–Co(1)–N(1)	87.73(18)	O(7)ii–Co(1)–O(1)	99.41(15)	O(7)ii–Co(1)–O(10)	162.46(16)
O(9)i–Co(1)–O(7)ii	87.35(15)	O(7)ii–Co(1)–N(1)	89.11(17)	O(7)ii–Co(1)–O(6)ii	59.43(14)
O(11)–Co(2)–O(2)iii	92.76(18)	O(11)–Co(2)–O(8)i	91.80(17)	O(8)i–Co(2)–O(2)iii	174.94(18)

O(11)–Co(2)–O(7)i	175.04(17)	O(7)i–Co(2)–O(2)iii	90.74(15)	O(7)i–Co(2)–O(8)i	84.58(15)
O(11)–Co(2)–O(3)	87.58(17)	O(2)iii–Co(2)–O(3)	90.29(15)	O(8)i–Co(2)–O(3)	87.71(16)
O(7)i–Co(2)–O(3)	88.90(15)	O(11)–Co(2)–N(2)	87.22(19)	O(2)iii–Co(2)–N(2)	94.59(17)
O(8)i–Co(2)–N(2)	87.83(17)	O(7)i–Co(2)–N(2)	96.01(17)	O(3)–Co(2)–N(2)	173.03(18)

Symmetry codes: (**1**) i: *x*–1, *y*, *z*; (**2**) i: –*x*+2, –*y*+1, –*z*+1; (**3**) i: *x*–1, *y*, *z*; ii: *x*–1, –*y*+1/2, *z*+1/2; iii: *x*, –*y*+1/2, *z*–1/2.

Table S5. Hydrogen bonds in crystal packing [Å, °] for **1–3**.

Compound	D–H···A	*d*(D–H)	*d*(H···A)	*d*(D···A)	∠DHA	Symmetry code
**1**	O(10)–H(1W)···O(6)	0.820	2.033	2.797	154.85	
	O(10)–H(2W)···O(4)	0.860	1.976	2.835	177.75	–*x*, –*y* + 1, –*z*
	O(11)–H(3W)···O(4)	0.850	2.103	2.953	179.30	*x* + 1, *y*, *z*
	O(11)–H(4W)···O(6)	0.850	2.119	2.969	179.64	
	O(12)–H(5W)···O(11)	0.850	2.026	2.876	179.29	–*x* + 1, –*y* + 1, –*z* + 1
	O(12)–H(6W)···O(4)	0.850	2.115	2.965	179.56	*x* +1, *y*, *z* + 1
2	O(10)–H(1W)···O(10)	0.695	2.343	2.867	133.62	–*x* + 2, –*y* + 2, –*z*
	O(10)–H(2W)···O(2)	0.851	2.420	2.867	113.45	–*x* + 2, –*y* + 2, –*z*
	O(11)–H(3W)···O(8)	0.851	1.903	2.689	152.95	–*x* + 2, –*y* + 2, –*z* + 1
	O(11)–H(4W)···O(12)	0.852	1.996	2.649	132.74	*x*, *y* + 1, *z*
	O(12)–H(5W)···O(3)	0.850	2.083	2.833	146.97	–*x* + 2, –*y* + 1, –*z*
	O(12)–H(6W)···O(6)	0.850	1.906	2.749	170.98	*x* – 1, *y*, *z*
**3**	O(10)–H(10A)···O(8)	0.911	1.896	2.697	145.53	*x* – 1, *y*, *z*
	O(10)–H(10B)···O(14)	0.906	1.942	2.807	159.21	–*x* + 1, –*y*, –*z* + 1
	O(11)–H(11A)···O(13)	0.875	2.124	2.754	128.35	–*x* + 1, –*y*, –*z* + 1
	O(11)–H(11B)···O(4)	0.874	1.816	2.639	156.20	
	O(12)–H(12A)···O(6)	0.850	2.006	2.812	157.89	*x* – 1, –*y* + 1/2, *z* + 1/2
	O(12)–H(12B)···O(14)	0.850	2.158	2.950	155.01	–*x* + 1, –*y*, –*z* + 1
	O(13)–H(13A)···O(1)	0.850	2.259	3.109	179.39	
	O(13)–H(13B)···O(4)	0.850	2.298	2.855	123.41	
	O(14)–H(14A)···O(12)	0.850	1.995	2.844	179.20	*x* + 1, *y*, *z*
	O(14)–H(14B)···O(4)	0.851	1.957	2.806	175.69	
